# Layer-dependent excellent thermoelectric materials: from monolayer to trilayer tellurium based on DFT calculation

**DOI:** 10.3389/fchem.2023.1295589

**Published:** 2023-10-12

**Authors:** Kexin Zhang, Rennong Yang, Zhehao Sun, Xihao Chen, Sizhao Huang, Ning Wang

**Affiliations:** ^1^ Air Traffic Control and Navigation College, Air Force Engineering University, Xi’an, China; ^2^ Research School of Chemistry, Australian National University, Canberra, ACT, Australia; ^3^ School of Materials Science and Engineering, Chongqing University of Arts and Sciences, Chongqing, China; ^4^ School of Science, Harbin University of Science and Technology, Harbin, China; ^5^ Key Laboratory of High-Performance Scientific Computation, School of Science, Xihua University, Chengdu, China; ^6^ State Key Laboratory of Precision Spectroscopy, East China Normal University, Shanghai, China

**Keywords:** layer-dependent, thermoelectric transport, tellurium, first-principles calculations, thermal transport

## Abstract

Monoelemental two-dimensional (2D) materials, which are superior to binary and ternary 2D materials, currently attract remarkable interest due to their fascinating properties. Though the thermal and thermoelectric (TE) transport properties of tellurium have been studied in recent years, there is little research about the thermal and TE properties of multilayer tellurium with interlayer interaction force. Herein, the layer modulation of the phonon transport and TE performance of monolayer, bilayer, and trilayer tellurium is investigated by first-principles calcuations. First, it was found that thermal conductivity as a function of layer numbers possesses a robust, unusually non-monotonic behavior. Moreover, the anisotropy of the thermal transport properties of tellurium is weakened with the increase in the number of layers. By phonon-level systematic analysis, we found that the variation of phonon transport under the layer of increment was determined by increasing the phonon velocity in specific phonon modes. Then, the TE transport properties showed that the maximum figure of merit (*ZT*) reaches 6.3 (p-type) along the *armchair* direction at 700 K for the monolayer and 6.6 (p-type) along the *zigzag* direction at 700 K for the bilayer, suggesting that the TE properties of the monolayer are highly anisotropic. This study reveals that monolayer and bilayer tellurium have tremendous opportunities as candidates in TE applications. Moreover, further increasing the layer number to 3 hinders the improvement of TE performance for 2D tellurium.

## 1 Introduction

Two-dimensional (2D) nanomaterials have become a hotspot in research since the discovery of graphene (Geim, 2009; Abergel et al., 2010), hexagonal BN (Meziani et al., 2015; Zhang et al., 2017a), transition metal dichalcogenides (TMDs) (Zhang et al., 2017b), and so on (Meziani et al., 2015; Wang et al., 2023; Zhang et al., 2023). This can be attributed to their distinctive layer-related and thermal properties (Gu et al., 2017). As a newly proposed type of 2D material, monoelemental 2D materials (ME2DMs) have application potential in many fields (Zhou et al., 2021). These elements are placed between non-metals and metals and have different allotropes with intersectional electronic and chemical properties (Si and Niu, 2020). Besides, ME2DMs containing single elements provide a suitable model for studying the mechanisms of tractable chemistries (Borlido et al., 2019). The success of graphene in 2004 demonstrated the prospects of ME2DMs and sparked research on other ME2DMs with excellent electrical, mechanical, thermal, and optical properties (Novoselov et al., 2004). The combination of electrons confined in the 2D honeycomb lattice and peculiar energy electronic properties endow graphene with extreme electronic mobility (Du et al., 2008; Huang et al., 2023) and transparency (Nair et al., 2008), thereby realizing quantum phenomena (Novoselov et al., 2007). In addition, other ME2DMs such as black phosphorus, arsenene, antimonene, and bismuthene have also been widely reported (Pumera and Sofer, 2017).

Due to unique properties, monolayer tellurium has been proven to have potential applications in robust piezoelectricity for reliable memory (Rao et al., 2022) and optical properties for all-optical non-linear photonic devices (Wu et al., 2019). Especially, the ultra-low thermal conductivity and excellent thermoelectric (TE) performance (Gao et al., 2018a; Sang et al., 2019) of monolayer tellurium have been predicted by simulation. However, up to now, studies on the thermal and TE properties of multilayer tellurium are lacking. Besides, the method of constructing heterojunctions provides an idea to regulate and control electronic (Bediako et al., 2018; Hamer et al., 2018), optoelectronic (Geim and Grigorieva, 2013; Withers et al., 2015), and thermal (Tielrooij et al., 2018) properties. These properties are achieved not only by monolayer but also by multilayer structures and are promising to be applied in nanoscale optoelectronic or electronic equipment. More importantly, previous reports have provided references to layer-dependent phonon transport and TE properties (Sun et al., 2019; Lee et al., 2021). However, the impacts of layer numbers on phonon transport and TE properties of multilayer tellurium have not been studied explicitly enough.

Inspired by the above, phonon transport and TE properties of monolayer, bilayer, and trilayer tellurium have been investigated by first-principles calcuations at 300 K in our study. We first discuss phonon dispersion and energy electronic properties of the three materials and further obtain and discuss differences in phonon thermal conductivity and TE transport properties among them. We provide theoretical predictions of layer-dependent thermal transport and TE transport properties through different layer numbers of tellurium. This unexpected discovery may provide some theoretical references for future research on the application of multilayer 2D materials.

## 2 Methodology

The first-principles calculations were completed within the method of density functional theory (DFT) using pseudopotentials as used in the Vienna *Ab initio* Simulation Package (VASP) code (Kresse and Furthmüller, 1996a; Kresse and Furthmüller, 1996b). The exchange-correlation energy function was treated using the Perdew-Burke-Ernzerhof (PBE) function of generalized gradient approximation (GGA) (Perdew et al., 1996). A kinetic energy cutoff of 500 eV was selected. The geometry-astringent tolerance for energy and force was less than 10^–8^ and 10^–6^ eV/Å. For all tellurium, the Monkhorst–Pack *k*-point grid was set to 13 × 13 × 1, and the vacuum region was set to 20 Å. The DFT-D3 methods for the vdW mechanism were also considered in our calculations. The interatomic force constants (IFCs) were obtained by the supercell (4 × 4 × 1 supercell) and *Γ*-point mesh method using the Phonopy (Togo et al., 2008). For the calculation of third-order IFCs in the thirdorder.py script (Li et al., 2014), a 4 × 4 × 1 supercell was also used. The cutoff was set for the 16th nearest number. The *Q*-grid of 60 × 60 × 1 was selected for obtaining the phonon properties. The lattice thermal conductivity (*κ*_*p*_) and phonon transport properties were calculated using ShengBTE (Li et al., 2014). In the electronic transport part, the electrical conductivity was calculated using BoltzTraP (Madsen and Singh, 2006). The carrier mobility (*μ*) was calculated using the deformation potential theory. In 2D systems, the carrier mobility can be expressed as (Qiao et al., 2014; Zhang et al., 2014)

$$\mu^{2D}=\frac{2{eh}^{3}C^{2D}}{3k_{B}T{\left|m^{*}\right|}^{2}E_{DP}},$$ (1)

where *C* ^*2D*^ is the elastic modulus, m* is the electronic effective mass, and *E*_*DP*_ is the deformation potential. In addition, the carrier relaxation time can be calculated by *τ* = μm*/e.

The performance of TE materials can be determined by the *ZT* value (Zhao et al., 2014):

$$ZT=\frac{S^{2}\sigma T}{\left(\kappa_{e}+\kappa_{p}\right)},$$ (2)

where *S*, *T*, *σ*, $\kappa_{e}$ , and $\kappa_{p}$ are the Seebeck coefficient, temperature, electrical conductivity, and electronic and phonon thermal conductivity, respectively. The electronic conductivity can be calculated as $\kappa_{e}=L\sigma T$ (Lorentz constant *L* = 1.5 × 10^−8^ WΩ/K^2^) (Jonson and Mahan, 1980; Stojanovic et al., 2010).

## 3 Discussion

### 3.1 Geometrical, phonon, and electronic structures

**FIGURE 1:**
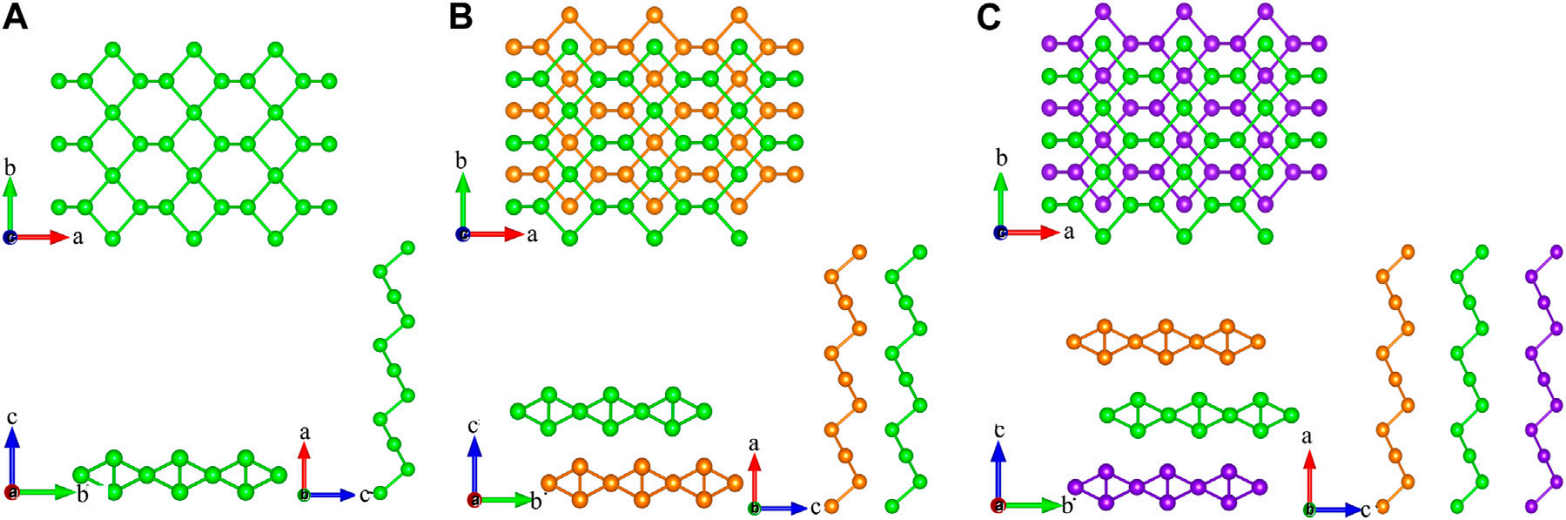
Crystal structures of the top and side views for **(A)** monolayer, **(B)** bilayer, and **(C)** trilayer tellurium.

As shown in Figures 1A–C, monolayer tellurium consists of three atomic subplanes with buckling distances, while graphene has a planar structure. The calculated lattice constant of monolayer tellurium by us in the *a* and *b* directions is 5.62 and 4.23 Å, respectively. These values are very close to previously calculated values of 5.69 and 4.23 Å by Sang et al. (2019) and other reports (Gao et al., 2018a; Gao et al., 2018b). Different lattice constants in different directions usually indicate the directional dependence of the transport properties. Bilayer tellurium built on an AA stack and trilayer tellurium built on an AAA stack has the same characteristics as monolayer tellurium. As shown in Figures 1B, C, the stacking methods of bilayer and trilayer for our structures are AB and ABA types, respectively. The formation of dislocations between layers significantly affects their physical and chemical properties and further leads to differences in their TE performance.

**FIGURE 2:**
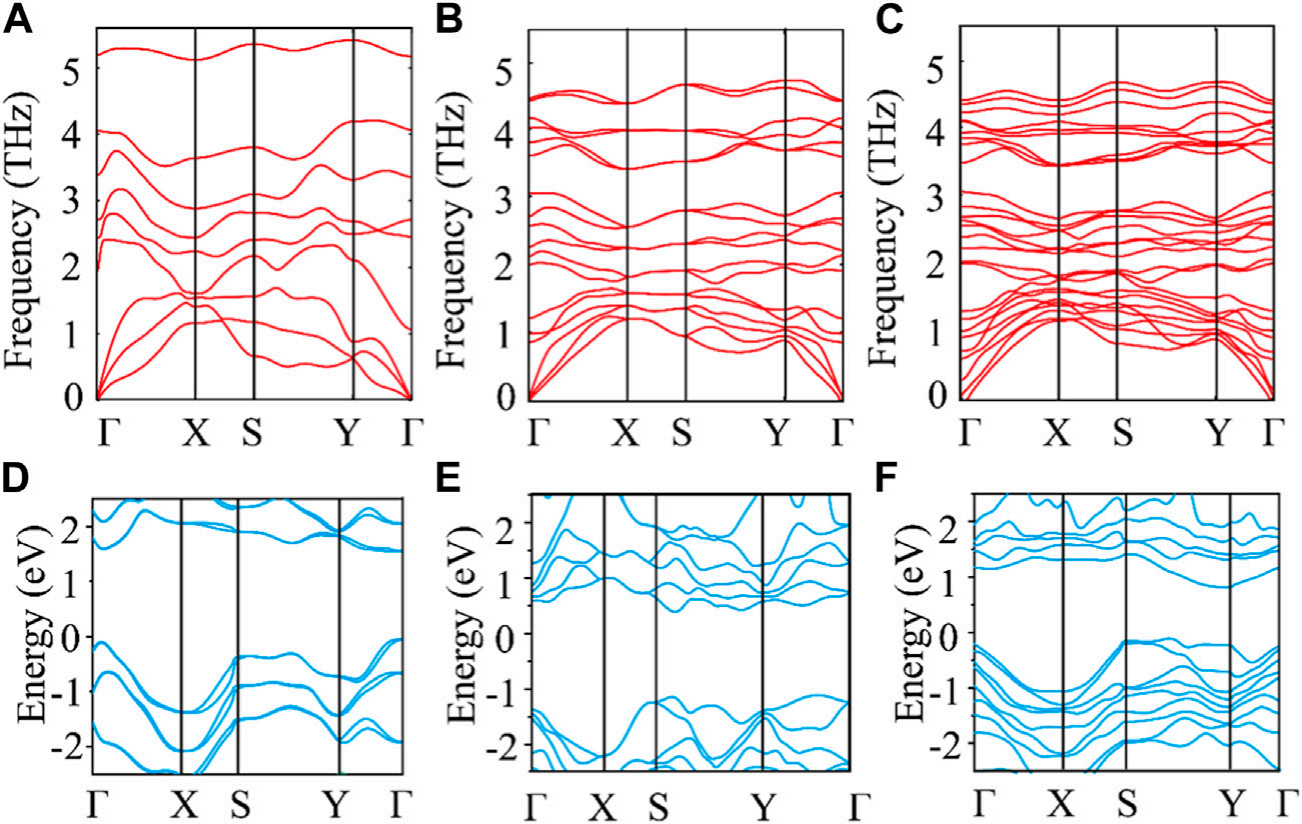
Phonon dispersion for **(A)** monolayer, **(B)** bilayer, and **(C)** trilayer tellurium. Electronic band structure for **(D)** monolayer, **(E)** bilayer, and **(F)** trilayer tellurium.

Figures 2A–C show the phonon spectra of monolayer, bilayer, and trilayer tellurium. The frequency of the phonon spectra of the three structures is greater than 0, confirming the stability of the material. Through the three figures, we can also see that the frequencies of the acoustic branch of tellurium and some optical branches near the acoustic branch are very small, much smaller than those of many 2D materials (Geim, 2009; Abergel et al., 2010). The low-frequency distribution is similar to that reported for ultralow thermal conductivity 2D materials, such as triphosphides (Sun et al., 2020), monolayer Hf_2_Cl_4_ (Li et al., 2021), and XIS (X = Al, Ga, In) (Cheng et al., 2022). Our results show that the phonon harmonic vibration of tellurium is very weak with a small thermal conductivity, as is expected of a TE material with good application potential. Obviously, the increasing number of layers can reduce the vibration frequency of the optical branch but does not affect the frequency distribution of the acoustic branch. In addition, we find that multilayer tellurium has an indirect energy gap and significant asymmetry between the conduction and valence bands. Moreover, the number of layers can effectively regulate the electronic band structure of tellurium. Specifically, the indirect bandgap of tellurium decreases with an increasing number of layers, as shown in Figures 2D–F. The bandgaps of monolayer, bilayer, and trilayer tellurium are ∼1.7, ∼1.5, and ∼0.9 eV, respectively. The bandgap of monolayer tellurium is very close to the previous report of ∼1.5 eV by Sang et al. (2019).

### 3.2 Thermal transport properties

**FIGURE 3:**
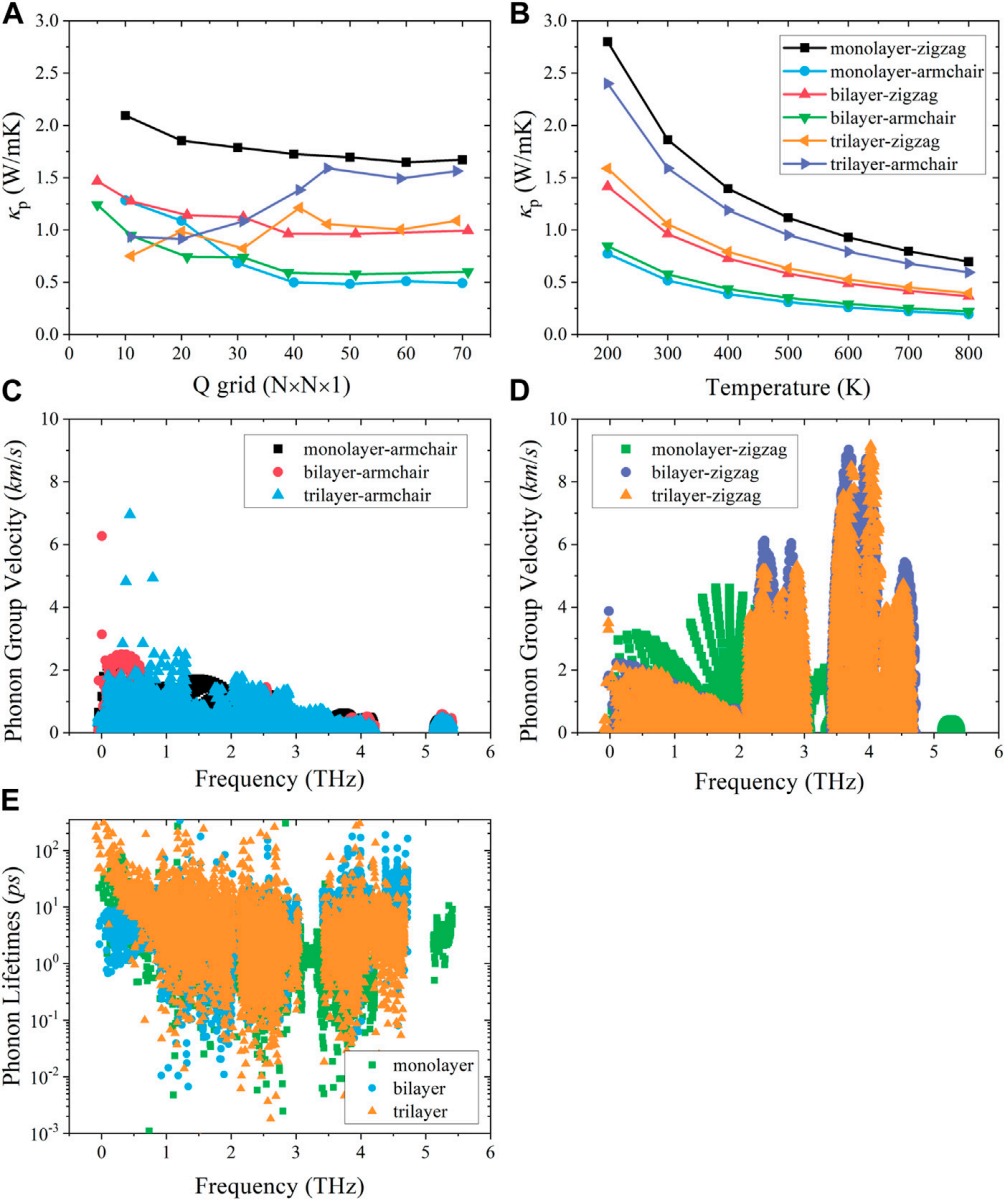
**(A)** Lattice thermal conductivity convergence test of the Q grid. **(B)** Lattice thermal conductivity of tellurium with different layer numbers at different temperatures along the *zigzag* and *armchair* directions. **(C)** Phonon group velocity along the *armchair* direction and **(D)** *zigzag* direction. **(E)** Phonon relaxation time.

In Figure 3A, we test the convergence of thermal conductivity for three materials along different directions. It can be seen that the thermal transport shows good convergence when the Q grids exceed 50. The results in Figure 3B show that the thermal conductivity of monolayer, bilayer, and trilayer tellurium follows the 1/T law with temperature, and the layer-dependent thermal transport properties have obvious differences. The trend of thermal conductivity with the number of layers is an unusual non-monotonic behavior. Most importantly, as the number of layers increases, there is an unexpected difference in thermal conductivity between different numbers of layers. That is, the thermal conductivity of monolayer tellurium along the *zigzag* direction is much larger than that of bilayer and trilayer tellurium (twice), while the thermal conductivity of bilayer and trilayer tellurium is similar along the *zigzag* direction. However, in the *armchair* direction, both monolayer and bilayer tellurium exhibit ultralow thermal conductivity, while the thermal conductivity of trilayer tellurium is the highest relative to single and double layers. The lattice thermal conductivity depends, to some extent, on phonon anharmonicity. The phonon group velocity can be determined by the slope of the phonon dispersion curve. Since the acoustic branch contributes the most to thermal transport, here, the only focus is on the acoustic branch phonon. The detailed analysis of the phonon harmonicity and anharmonicity of the different layers of tellurium is as follows: the group velocities of phonons with high frequency along the *armchair* direction are similar, with the main difference being at low frequencies of 0–2 THz (Figure 3C). The values of the monolayer are the smallest at low frequencies, resulting in the lowest phonon thermal conductivity. The phonon group velocity of the bilayer and trilayer is similar. For the *zigzag* direction, in the 0–2 THz region (Figure 3D), the magnitude and distribution of the phonon group velocities for the bilayer and trilayer are similar, while the monolayer is significantly larger than the bilayer and trilayer, so the phonon transport of the monolayer along the *zigzag* direction is the strongest. We also analyzed the impact of layer number on anharmonicity. We find that interlayer interactions weaken the anharmonicity. In particular, trilayers exhibit higher phonon relaxation times at low frequencies (Figure 3E), and surface interlayer interactions weaken intrinsic phonon–phonon interaction and lead to the enhancement of anharmonicity.

### 3.3 Electrical transport properties

As shown in Table 1, we have predicted the $m_{b}^{*}=m*/m_{0}$ , *E*_*DP*_, *C* ^*2D*^, and *μ* of the monolayer, bilayer, and trilayer tellurium at room temperature with different types of doping along the *zigzag* and *armchair* directions through the first-principles calculations (Qiao et al., 2014; Zhang et al., 2014). For the monolayer, the anisotropy of the *in-plane C* ^*2D*^ can be ignored, and other parameters have obvious anisotropy in the *armchair* and *zigzag axes* of our unit cell. Owing to the effective mass of the holes in the monolayer being very small, a high carrier mobility of 0.209 m^2^ V^−1^s^−1^ for p-type doped tellurium along the *armchair* direction can be calculated. The bilayer also shows anisotropic electronic properties, but the $m_{b}^{*}$ is relatively small and has a higher carrier mobility and electron relaxation time. Although electrons have a high *μ* for trilayer, the p-type doping has a larger $m_{b}^{*}$ , and the *μ* and *τ* are not very high. Through the calculation results in Table 2, the variation of electron relaxation time (*τ*) with temperature is obtained, and the TE transport properties are further obtained.

**TABLE 1:** Effective mass m*/m_*0*_, DP constant *E*_*DP*_, elastic constant *C* ^*2D*^ *,* and carrier mobility *μ* of monolayer, bilayer, and trilayer tellurium at room temperature.

		m*/m_*0*_	*E*_*DP*_ (eV)	*C* ^*2D*^ (N m^-1^)	*μ* (m^2^ V^−1^s^-1^)
Monolayer					
p-type	h-zigzag	0.434	4.697	30.698	0.011
p-type	h-armchair	0.240	1.965	32.604	0.209
n-type	e-zigzag	1.131	2.934	30.698	0.004
n-type	e-armchair	0.627	4.942	32.604	0.005
Bilayer					
p-type	h-zigzag	1.260	1.121	50.965	0.036
p-type	h-armchair	0.380	4.133	70.040	0.040
n-type	e-zigzag	0.698	2.735	50.965	0.020
n-type	e-armchair	0.161	5.745	70.040	0.117
Trilayer					
p-type	h-zigzag	1.587	2.745	79.266	0.006
p-type	h-armchair	0.879	3.453	87.900	0.014
n-type	e-zigzag	0.642	1.270	79.266	0.190
n-type	e-armchair	0.356	1.625	87.900	0.376

**TABLE 2:** Relaxation time *τ* (*f*s) of monolayer, bilayer, and trilayer tellurium at 300, 500, and 700 K.

			300 K	500 K	700 K
Monolayer	p-type	h-zigzag	25.985	15.591	11.137
	p-type	h-armchair	285.080	171.048	122.177
	n-type	e-zigzag	25.523	15.314	10.938
	n-type	e-armchair	17.215	10.329	7.378
Bilayer	p-type	h-zigzag	260.423	156.254	111.610
	p-type	h-armchair	87.311	52.387	37.419
	n-type	e-zigzag	79.098	47.459	33.899
	n-type	e-armchair	106.795	64.077	45.769
Trilayer	p-type	h-zigzag	53.708	32.225	23.018
	p-type	h-armchair	67.810	40.686	29.062
	n-type	e-zigzag	692.384	415.430	296.736
	n-type	e-armchair	759.934	455.960	325.686

**FIGURE 4:**
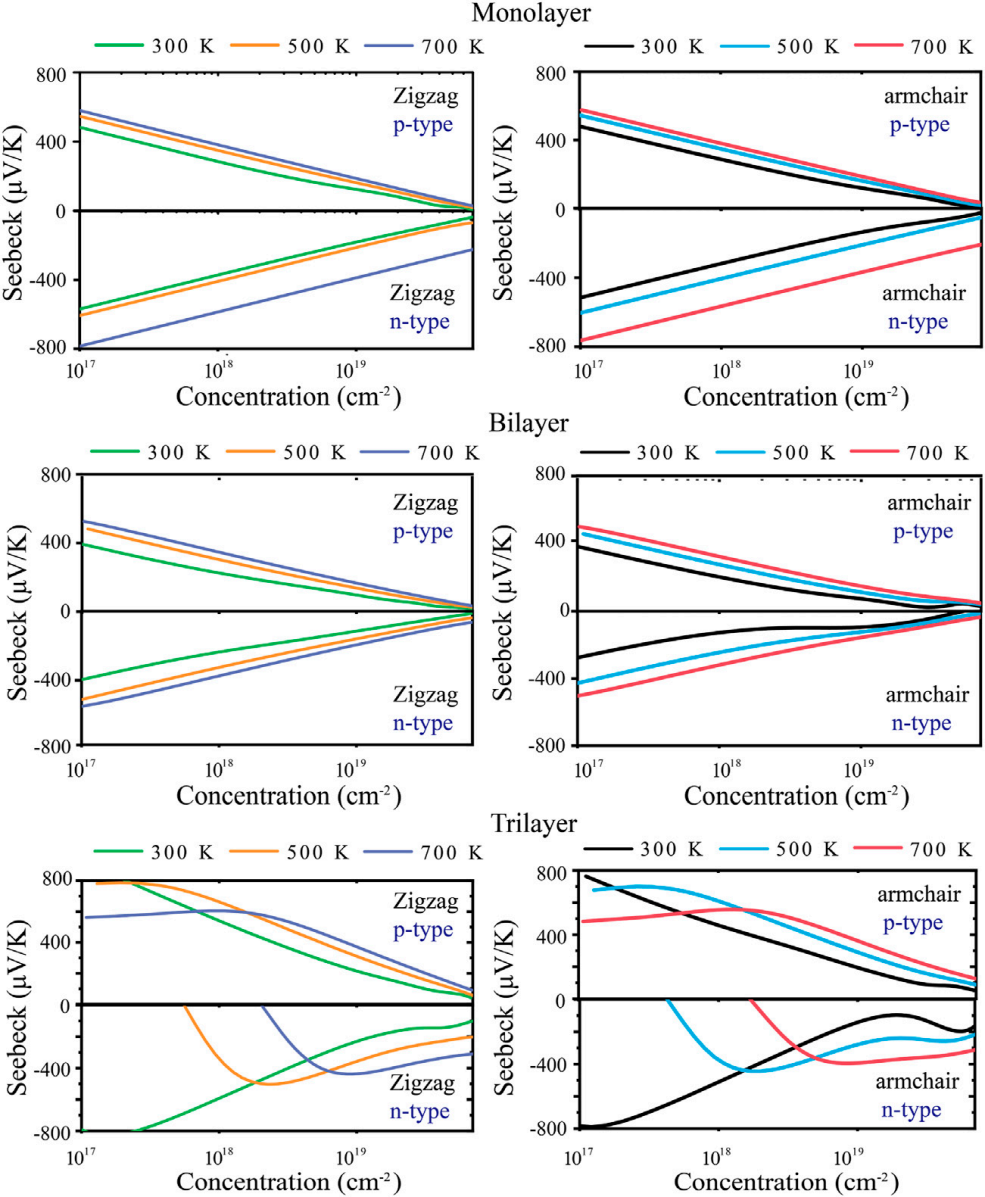
Calculated Seebeck coefficient *S* along *zigzag* and *armchair* directions at 300, 500, and 700 K.

**FIGURE 5:**
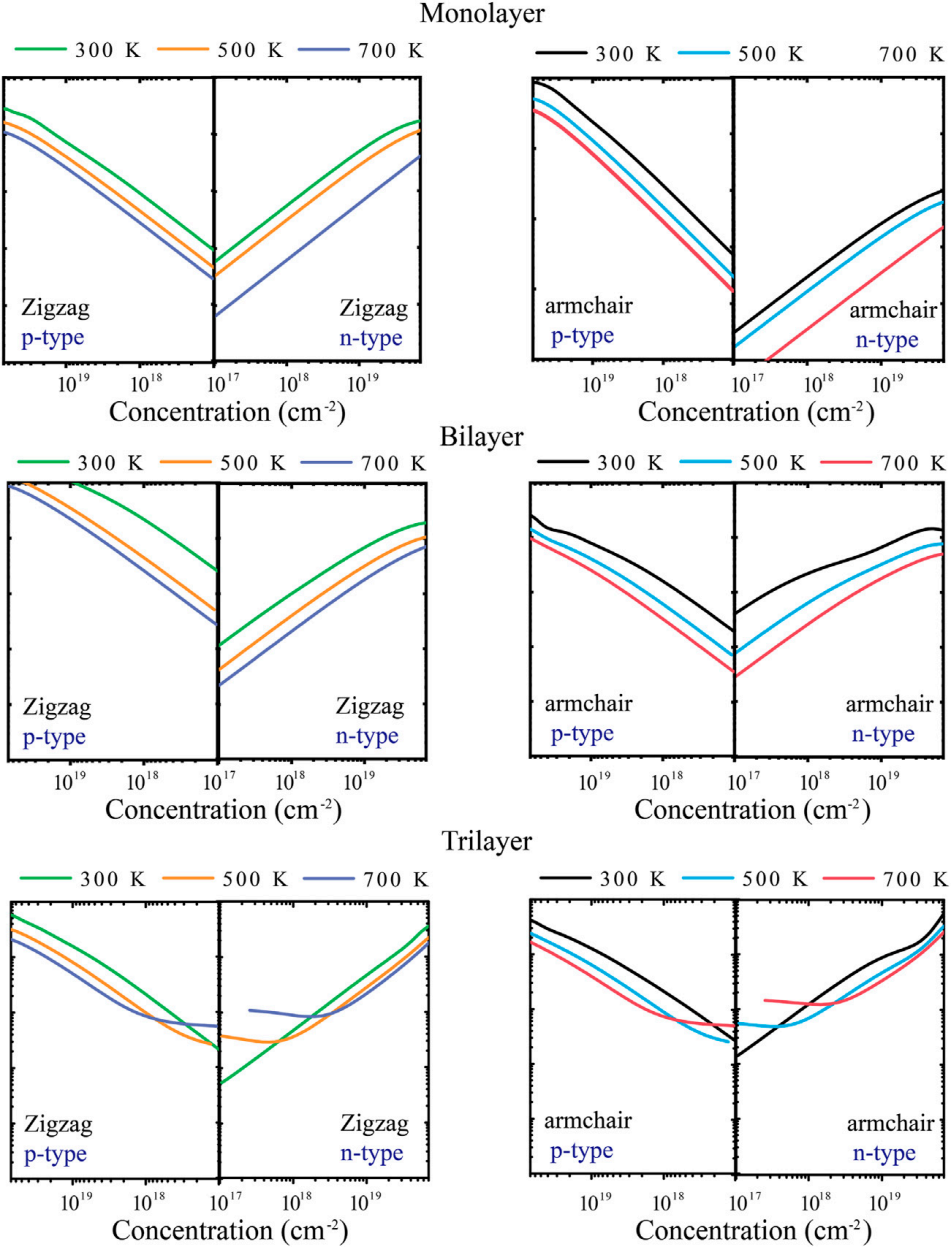
Calculated electrical conductivity *σ* along *zigzag* and *armchair* directions at 300, 500, and 700 K.

The energy conversion efficiency of the TE materials can be assessed with the dimensionless figure of merit (*ZT*). The TE transport properties of tellurium with different layers are mainly explored. Figure 4 shows the calculated *S* of n-type and p-type doped tellurium with different layers as a function of the carrier concentration at 300, 500, and 700 K. The monolayer has anisotropic *S*. It can be found that *S* increases with the carrier concentration along the *zigzag* and *armchair* directions, almost similar to the bilayer. This phenomenon indicates that *S* is isotropic for bilayer tellurium, and *S* of n-type doped monolayer tellurium can reach ∼600 μV/K at a carrier concentration of 1 × 10^11^ cm^-2^ under 300 K, while that of n-type doped bilayer tellurium can reach ∼400 μV/K. When the environmental temperature reaches 700 K, *S* becomes larger. In fact, the *S* coefficient of typical TE materials is only between 200 and 300 μV/K, while the *S* coefficient of monolayer and multilayer tellurium is much better than that of the mature TE material SnSe (Guo et al., 2015). High values of *S* indicate that higher *ZT* may exist in the tellurium system. The origin of very high *S* values is related to the previously mentioned band structure. In Figure 1, the band graph has a multi-valley characteristic, which leads to a high slope of the density of states (DOS) near the Fermi energy, resulting in a high *S*. For monolayer n-type doped tellurium, *S* is greater than that of p-type tellurium at an effective carrier concentration *n*, mainly because the number of conduction band energy valleys is greater than the valence band. Contrary to *S*, *σ* increases with an increase in *n*, as shown in Figure 5. *σ* changes little at different temperatures, and bilayer and trilayer tellurium have their respective weaker anisotropies. When the carrier concentration increases, the *σ* of p-type doping is greater than that of n-type doping in monolayer and bilayer tellurium. However, the trilayers have similar electrical conductivities under the two different doping types. As we know, *σ* is directly proportional to the contributed *κ*_*e*_, which means that the greater the *σ*, the higher the electronic thermal conductivity *κ*_*e*_. In fact, TE properties not only depend on *σ*. This is because *S*, *σ,* and *κ*_*e*_ are interrelated in TE transport. In this work, using the abovementioned calculated TE transport parameters, we have predicted the TE performance for tellurium with different layers.

### 3.4 Figure of merit

**FIGURE 6:**
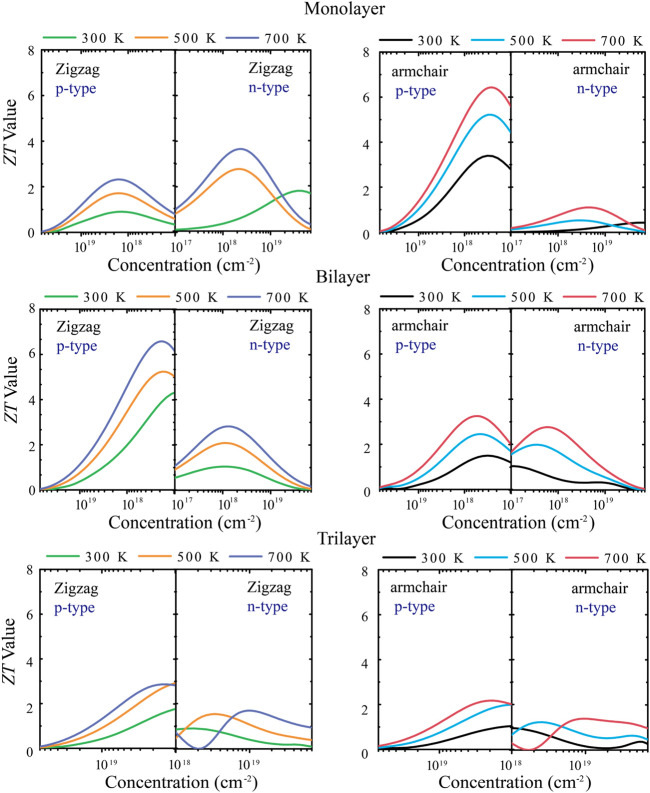
Calculated dimensionless figure of merit (*ZT*) along *zigzag* and *armchair* directions at 300, 500, and 700 K.

Figure 6 shows the relationship between the *ZT* value with different layers of tellurium and the carrier concentration under different types of doping. First of all, for the monolayer, the maximum *ZT* value of p-type doping along the *armchair* direction is 6.3 at a lower carrier concentration (10^11^–10^12^ cm^−2^). For the *zigzag* direction, the *ZT* value is not very high, so the p-type doping has a strong anisotropy. For n-type doping, the *ZT* value reaches 3.8 in the *zigzag* direction under 700 K at lower carrier concentrations (10^11^–10^12^ cm^−2^). For the bilayer, the *ZT* value along the *armchair* is not high, and the main reason is that the bilayer has stronger phonon thermal transport along the *armchair* direction. Its p-type doping has strong anisotropy, and the *ZT* value can reach 6.6 under 700 K along the *zigzag* direction at low carrier concentrations (10^11^–10^12^ cm^−2^). Such a high *ZT* value of bilayer tellurium is also larger than other typical 2D TE materials, such as ∼0.8 at 700 K for Bi_2_Te_3_ monolayer (Rashid et al., 2019), 3.46 at 500 K for SnP_3_ monolayer (Zhu et al., 2019), and 3.45 at 800 K for Bi_2_O_2_Se monolayer (Wang et al., 2019). For trilayer tellurium, the anisotropy of the *ZT* value is weakened, and the *ZT* value is lower than it is for monolayer and bilayer tellurium. The abovementioned calculations indicate that monolayer and bilayer tellurium are p-type TE materials with high *ZT* values and have broad practical application expectations in TE and energy-related applications in the future. When the structure has three layers, the TE performance of tellurium will decrease.

## 4 Conclusion

In this study, based on the Boltzmann transport equation and first-principles calculations, the TE transport parameters of monolayer, bilayer, and trilayer tellurium materials were comprehensively explored and compared. First, our results validate their thermal stability and determine the structural reliability by calculating the atomic second-order force constants. The three abovementioned 2D tellurium materials all have low lattice thermal conductivity. The lattice thermal conductivity does not change monotonically with the number of layers. Through analysis of phonon group velocity and relaxation time, it has been explained that phonon harmonicity dominates thermal conductivity. Unlike the phenomenon where the effective mass of holes in bilayer and trilayer structures is greater than that of electrons, for monolayer structures, the $m_{b}^{*}$ of the electrons is higher than that of the holes. This leads to low carrier mobility, and the *σ* of the n-type monolayer tellurium becomes lower than that of the p-type doped system. With the abovementioned results and *S*, the relationship between the *ZT* values at different temperatures as a function of carrier concentration is obtained. The maximum *ZT* value of monolayer, bilayer, and trilayer tellurium can reach 6.6, 6.3, and 3.8, respectively. Due to weak phonon transport, high *S*, and high *μ* of monolayer/bilayer tellurium, these exhibit excellent TE properties. Although trilayer tellurium has low thermal conductivity, the *ZT* values are not as high as those of monolayer and bilayer tellurium.

## Data Availability

The original contributions presented in the study are included in the article/Supplementary Material. Further inquiries can be directed to the corresponding authors.
